# Correction: Developing climate-resilient rice varieties (BRRI dhan97 and BRRI dhan99) suitable for salt-stress environments in Bangladesh

**DOI:** 10.1371/journal.pone.0317153

**Published:** 2025-01-03

**Authors:** Sanjoy K. Debsharma, M. Akhlasur Rahman, Mahmuda Khatun, Ribed F. Disha, Nusrat Jahan, Md. Ruhul Quddus, Hasina Khatun, Sharifa S. Dipti, Md. Ibrahim, K. M. Iftekharuddaula, Md. Shahjahan Kabir

The images for Figs 1 and 2 are incorrectly switched. The image that appears as Fig 1 should be Fig 2, and the image that appears as Fig 2 should be Fig 1. The figure captions appear in the correct order.

**Fig 1:**
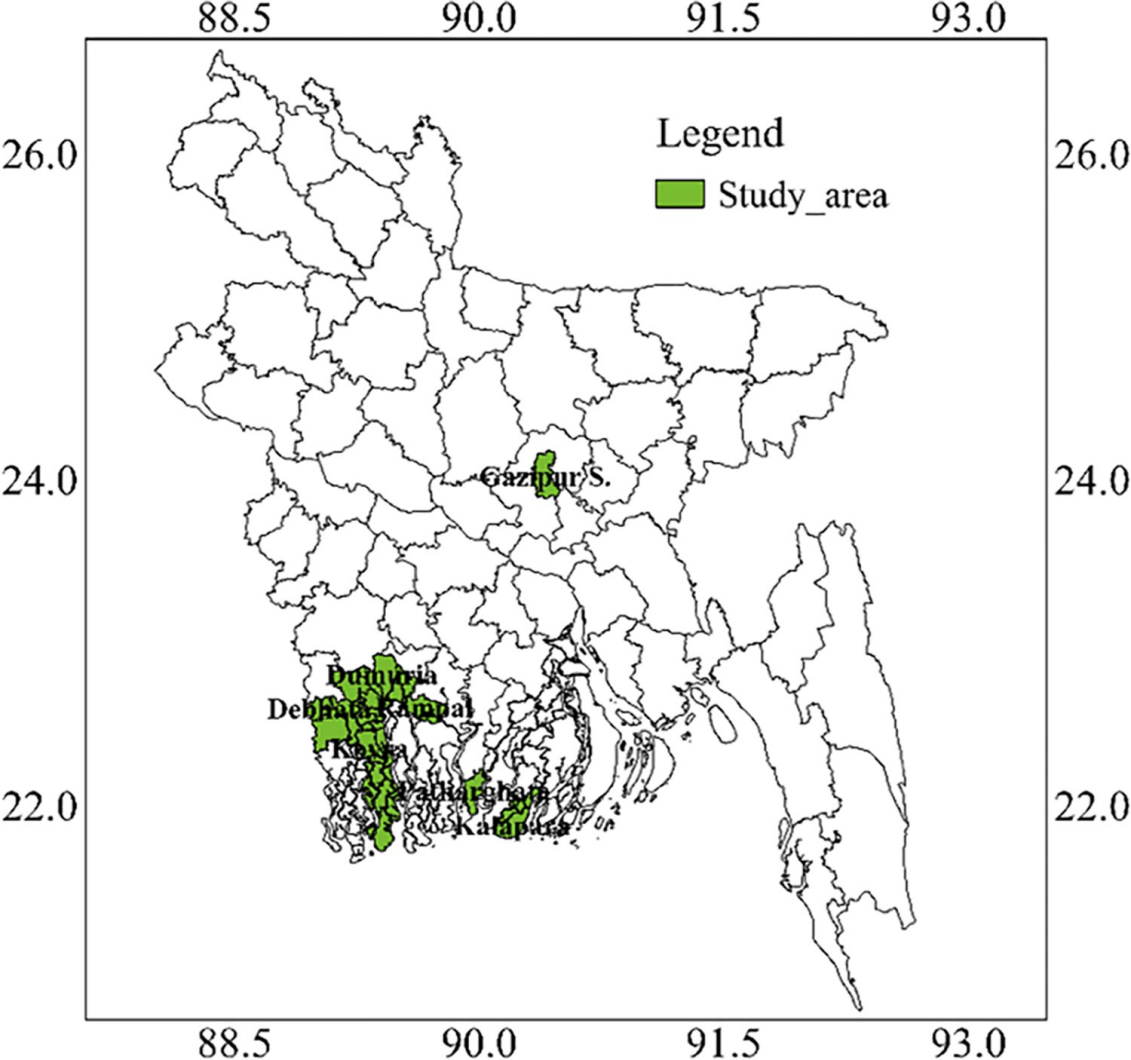
Geographic illustration of the different experimental locations across Bangladesh. The green color indicates the studied area where trials were conducted. Most studied areas were located in the southern coastal regions of Bangladesh, near the Bay of Bengal is the northeastern part of the Indian Ocean.

**Fig 2:**
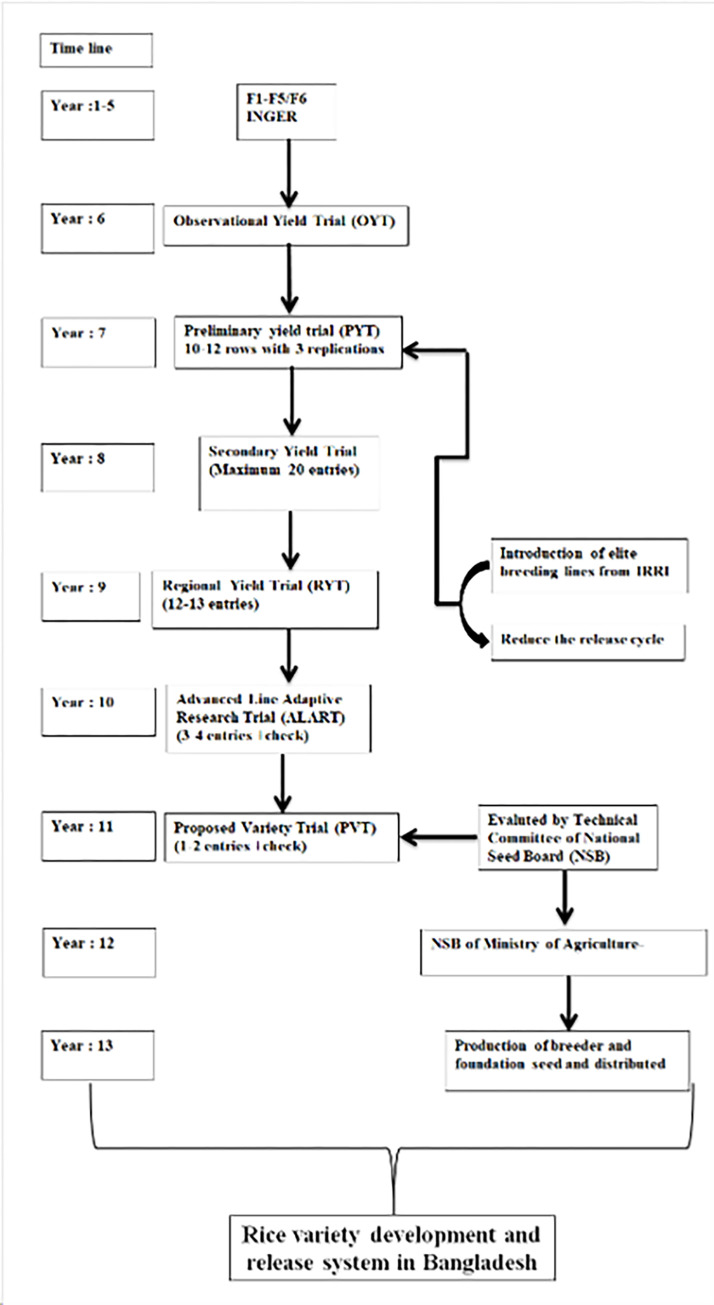
Variety release system for BRRI developed breeding lines and exotic introduced advanced breeding materials in Bangladesh.
