# Supplementary material for: Developing climate-resilient rice varieties (BRRI dhan97 and BRRI dhan99) suitable for salt-stress environments in Bangladesh
Source: PLoS One. 2024 Jan 19;19(1):e0294573. doi: 10.1371/journal.pone.0294573 (PMC10810675; doi:10.1371/journal.pone.0294573)
Supplement: S1 Fig — (PDF) [file pone.0294573.s001.pdf]

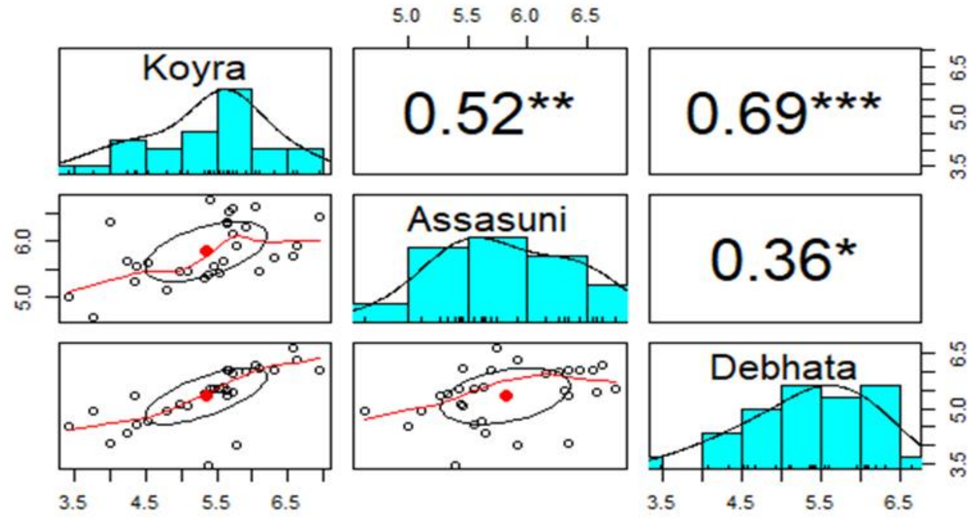

S1 Fig. Correlation of three locations for yield performance in the Regional Yield Trial (RYT) during Boro 2016-17. The correlation coefficient and the level of significance are displayed as stars at the top of the diagonal. * $p \leq 0.05$ and ** $p \leq 0.01$ show significance level.
