# Supplementary material for: Developing climate-resilient rice varieties (BRRI dhan97 and BRRI dhan99) suitable for salt-stress environments in Bangladesh
Source: PLoS One. 2024 Jan 19;19(1):e0294573. doi: 10.1371/journal.pone.0294573 (PMC10810675; doi:10.1371/journal.pone.0294573)
Supplement: S2 Fig — The degree of salinity was found highest at Kaliganj and the lowest at Tala, Satkhira in farmers’ fields. (PDF) [file pone.0294573.s002.pdf]

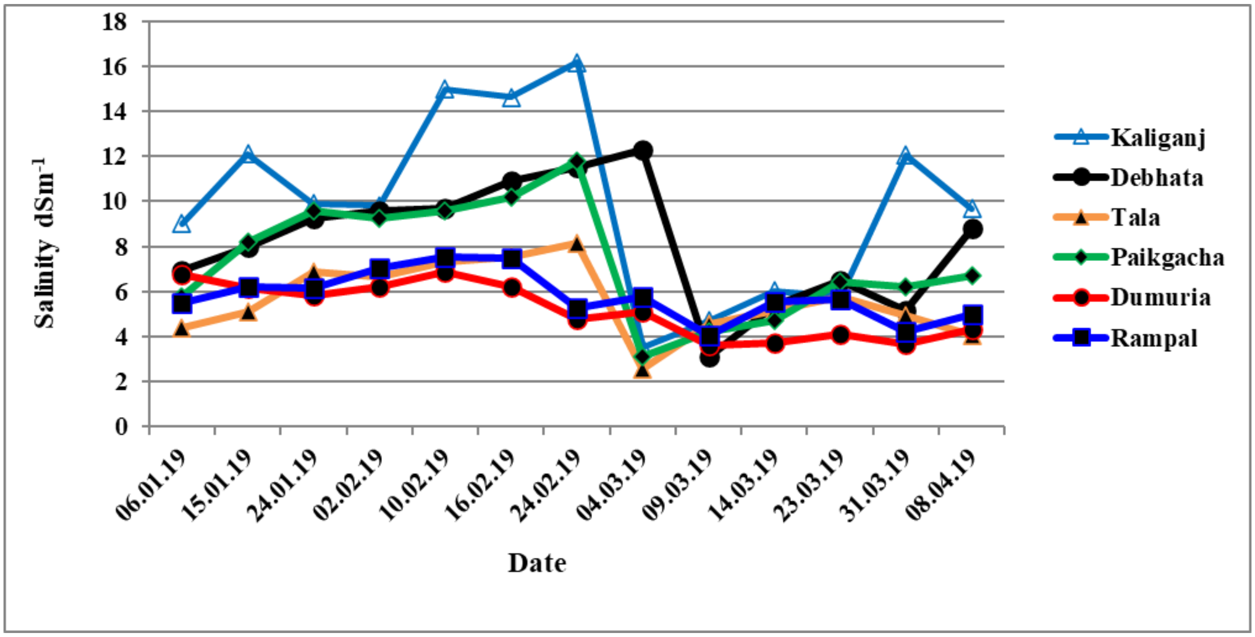

S2 Fig. The salinity level of the proposed variety trial varied at different locations of the coastal zone during the Proposed Variety Trial during Boro2018-19. The degree of salinity was found highest at Kaliganj and the lowest at Tala, Satkhira in farmers' fields.
