# Supplementary material for: Developing climate-resilient rice varieties (BRRI dhan97 and BRRI dhan99) suitable for salt-stress environments in Bangladesh
Source: PLoS One. 2024 Jan 19;19(1):e0294573. doi: 10.1371/journal.pone.0294573 (PMC10810675; doi:10.1371/journal.pone.0294573)
Supplement: S3 Fig — a) Unrooted neighbour-joining tree and b) an UPGMA (unweighted pair group method with arithmetic mean) cluster dendrogram showing genetic relationships between the four proposed lines (IR83484-3-B-7-1-1-1: BRRI dhan97, BR9011-67-4-1: BRRI dhan98, HHZ5-DT20-DT2-DT1: BRRI dhan99, HHZ12-SAL2-Y3-Y2 and three check varieties (BR26, BRRI dhan28, and BRRI dhan67) based on the 1K Rice Custom Amplicon assay or 1k-RiCA SNP markers. All four lines and three checks showed distinct molecular variations/differences among them. (PDF) [file pone.0294573.s003.pdf]

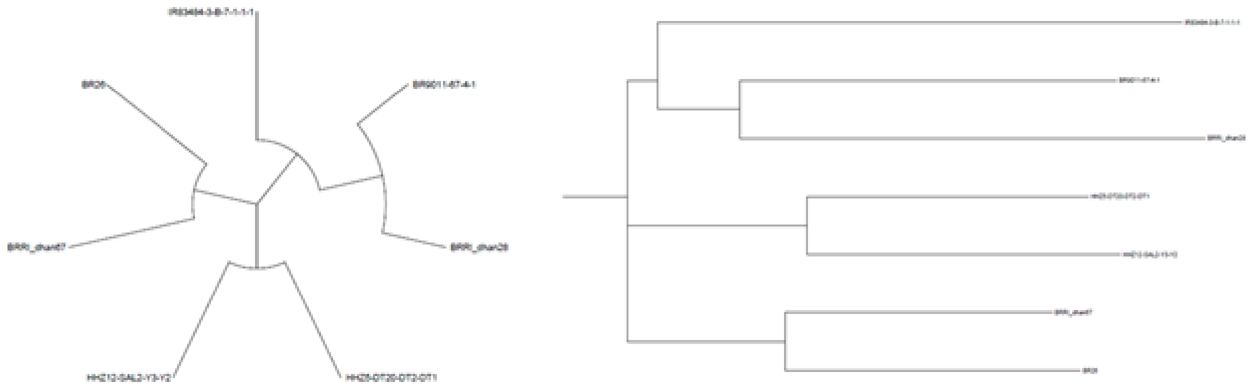

S3 Fig. a) Unrooted neighbour-joining tree and b) an UPGMA (unweighted pair group method with arithmetic mean) cluster dendrogram. It reveals genetic relationships between the four proposed lines (IR83484-3-B-7-1-1-1: BRRI dhan97, BR9011-67-4-1: BRRI dhan98, HHZ5-DT20-DT2-DT1: BRRI dhan99, HHZ12-SAL2-Y3-Y2 and three check varieties (BR26, BRRI dhan28 and BRRI dhan67) based on the 1K Rice Custom Amplicon assay or 1k-RiCA SNP markers. All the four lines and three checks showed distinct molecular variations/differences among them.
