# Supplementary material for: Developing climate-resilient rice varieties (BRRI dhan97 and BRRI dhan99) suitable for salt-stress environments in Bangladesh
Source: PLoS One. 2024 Jan 19;19(1):e0294573. doi: 10.1371/journal.pone.0294573 (PMC10810675; doi:10.1371/journal.pone.0294573)
Supplement: S4 Fig — YLD: Yield, DM: Days to maturity, PH: Plant height, ET: Effective tillers, PL: Panicle length, UFG: Unfilled grain. (PDF) [file pone.0294573.s004.pdf]

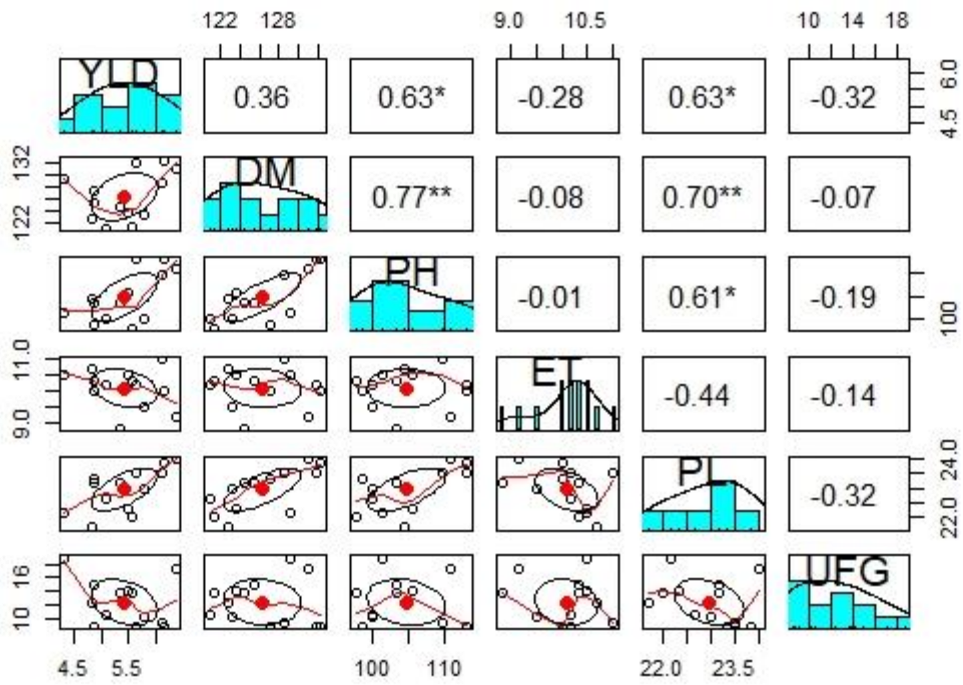

S4 Fig. Correlation between yield and yield-associated agronomic traits in the regional yield trial. YLD: Yield (t/ha), DM: Days to maturity (days), PH: Plant height (cm), ET: Effective tillers, PL: Panicle length (cm), UFG: Unfilled grain. The correlation coefficient and the level of significance are displayed as stars at the top of the diagonal. * $p \leq 0.05$ and ** $p \leq 0.01$ show significance level.
