# Supplementary material for: Developing climate-resilient rice varieties (BRRI dhan97 and BRRI dhan99) suitable for salt-stress environments in Bangladesh
Source: PLoS One. 2024 Jan 19;19(1):e0294573. doi: 10.1371/journal.pone.0294573 (PMC10810675; doi:10.1371/journal.pone.0294573)
Supplement: S1 Table — (PDF) [file pone.0294573.s005.pdf]

S1 Table. Participatory Varietal Selection of rice genotypes grown at Assasuni, Debhata, Kaliganj in Satkhira and Koyra in Khulna districts during Boro2016-17

Code	Designation	Assasuni	Debhata	Koyra	Kaligonj
PVS-1	BR8940-B-17-4-7				1st -ve
PVS-2	BR8943-B-20-9-22			1st -ve	2nd -ve
PVS-3	IR86385-85-2-1-B	1st -ve			
PVS-4	IR83484-3-B-7-1-1-1		1st +ve	2nd+ve	1st +ve
PVS-5	IR87872-7-1-1-2-1-B				
PVS-6	IR86385-117-1-1-B	2nd -ve	2nd -ve	2nd -ve	
PVS-7	IR87870-6-1-1-1-1-B				
PVS-8	BR8980-4-6-5				
PVS-9	BR8980-B-1-3-5				
PVS-10	BR8992-B-18-2-26		1st -ve		
PVS-11	HHZ5-DT20-DT2-DT1			1st +ve	2nd+ve
PVS-12	HHZ12-SAL2-Y3-Y2	1st +ve			
PVS-13	BRRI dhan28 (S. Ck)				
PVS-14	BRRI dhan67 (Ck)		2nd+ve		
PVS-15	Binadhan-10 (Ck)	2nd+ve			
