# Supplementary material for: Developing climate-resilient rice varieties (BRRI dhan97 and BRRI dhan99) suitable for salt-stress environments in Bangladesh
Source: PLoS One. 2024 Jan 19;19(1):e0294573. doi: 10.1371/journal.pone.0294573 (PMC10810675; doi:10.1371/journal.pone.0294573)
Supplement: S2 Table — (PDF) [file pone.0294573.s006.pdf]

S2 Table. Distinguish characterization of Distinctness, Uniformity and Stability (DUS) test for BRRI dhan97 and BRRI dhan99 based on Zadoks scale (Zadoks et al. 1974)

SN	Characteristics	BRRI dhan97		BRRI dhan28 (Ck.)		SN	Characteristics	BRRI dhan99		BRRI dhan28 (Ck.)	
		Code	State	Code	State			Code	State	Code	State
1	Leaf sheath: anthocyanin color	2	Present	1	Absent	7	Flag leaf: attitude of blade	3	Semi erect	5	Horizontal
7	Flag leaf: attitude of blade	3	Semi Erect	5	Horizontal	28	Panicle: exertion	9	Well exerted	5	Just
18	Stem: anthocyanin colouration of internodes	2	Present	1	Absent	31	Grain: length (without dehulling)	9	Very long	7	Long
33	Decorticated grain: length (After dehulling, before milling)	3	Medium	5	Long	-	-	-	-	-	-
35	Decorticated grain: shape (Length-breadth ratio)	7	Medium bold	9	Slender	-	-	-	-	-	-
40	Other distinct special character	Salinity tolerance		Salinity susceptible		40	Other distinct special character	Salinity tolerance		Salinity susceptible	
