# Supplementary material for: Developing climate-resilient rice varieties (BRRI dhan97 and BRRI dhan99) suitable for salt-stress environments in Bangladesh
Source: PLoS One. 2024 Jan 19;19(1):e0294573. doi: 10.1371/journal.pone.0294573 (PMC10810675; doi:10.1371/journal.pone.0294573)
Supplement: S3 Table — (PDF) [file pone.0294573.s007.pdf]

**S3 Table. Soli salinity classes and the ranges of Electrical Conductivity (EC) (Source: Soil Resource Development Institute, 2010)**

| Salinity class | EC ($dSm^{-1}$) |
|---|---|
| Non-saline to very slight saline | 2-4 |
| Very slight saline to slight saline | 4-8 |
| slight saline to moderate saline | 8-12 |
| Moderate saline to strong saline | 12-16 |
| strong saline to very strong saline | >16 |
