# Supplementary material for: Developing climate-resilient rice varieties (BRRI dhan97 and BRRI dhan99) suitable for salt-stress environments in Bangladesh
Source: PLoS One. 2024 Jan 19;19(1):e0294573. doi: 10.1371/journal.pone.0294573 (PMC10810675; doi:10.1371/journal.pone.0294573)
Supplement: Attachment — Submitted filename: Reviewer_2_comments and response.docx [file pone.0294573.s008.docx]

**Reviewer: 2_Comments and Suggestions for Authors**

Reviewer #2: The paper is very interesting and good piece of work from shortlisting of elite lines to release varieties for commercial cultivation. However, I found certain issues to be improved for its scientific accuracy and recommendations. There is no line number on MS, so difficult to prompt for specific parts of MS.

Response: Thank you for the encouraging comments and great suggestions.

Q1. There is no clue - how these 12 rice genotypes were selected? What was the basis or past research efforts to choose these 12 genotypes? Arbitrary?

Response: In the variety released system of Bangladesh, there are several steps to release a rice variety: At first the genotypes were selected by the Pedigree method (F2 to F6), then carried out Observational Yield Trial (OYT) followed by Preliminary Yield Trial (PYT), Regional Yield Trial (RYT), Advanced Line Adaptive Research Trial (ALART) and finally Proposed Variety Trial (PVT). The genotypes were selected based on yield, growth duration, and plant height compare checks. Genotypes G3, G4, G5, G6, G7, G11and G12 were introduced by International Rice Research Institute (IRRI), Los Banos, Philippines.

Q2. Please try to avoid grey sources to cite for big estimates. Do you feel that reference 11 is good enough (working on PGP) to refer to as prediction for 2050? Even reference 12 is a compilation from FAO and other sources. Please doubly check if the narrative is correct. Once satisfied for authenticity of source, then cite. Same issue for reference 4: "billions of hectares are effected by salinity? Is this correct? How many billion ha?; what percent of total arable area? You mentioned rice is being cultivated in 166 mha globally (not billion), how much of that under salinity?

Response: Of the total 14 billion ha of land available on earth, about 1 billion ha are natural saline soils. Also, it is estimated that worldwide about 20% of total cultivated lands and 33% of irrigated agricultural lands are afflicted by high salinity (Epstein et al., 1980; Shrivasata and Kumar, 2015).

Epstein, E., Norlyn, J. D., Rush, D. W., Kingsbury, R. W., Kelly, D. B., Gunningham, G. A. and Wrona, A. F. 1980. Saline culture of crops: A genetic approach. Science, 210: 399–404.

Shrivastava, P., & Kumar, R. (2015). Soil salinity: A serious environmental issue and plant growth promoting bacteria as one of the tools for its alleviation. *Saudi journal of biological sciences*, *22*(2), 123-131.

It has been estimated that more than 50% of the arable land would be salinized by the year 2050 (Jamil et al., 2011).

Jamil, A., S. Riaz, M. Ashraf, and M. R. Foolad. "Gene expression profiling of plants under salt stress." Critical Reviews in Plant Sciences 30, no. 5 (2011): 435-458.

Q3. Do you mean salt-responsive as quickly negatively affected by stress? Please clarify.

Response: Salt stress is a major environmental stress that affects plant growth and development. Salt stress increases the intracellular osmotic pressure and can cause the accumulation of sodium to toxic levels. Like other abiotic stresses, salt stress negatively affects plant growth and reproduction in many ways. Being a non-halophytic plant/glycophyte, the salt-responsiveness of rice is obviously negative, and quick destruction of the plant tissue (beyond its threshold level) has occurred during the seedling and reproductive stages.

Q4. Locations for RYT, ALART and PVT etc could be given in table along with stress ranges to understand the locations easily. (M & M 2.1)

Response: The table contains trial, locations along with water salinity status given below:

| Experiment/Trial | Location name | Status of water salinity |
| --- | --- | --- |
| RYT | Assasuni, Sathkira | Low stress |
|  | Kaliganj, Satkhira | High stress |
|  | Debhata, Satkhira | Low to high stress |
|  | Koyra, Khulna | Medium stress |
| ALART | BRRI Gazipur | No stress (Favorable) |
|  | Debhata, Satkhira | Low to high stress |
|  | Assasuni, Satkhira | Favorable |
|  | Batiaghata, Satkhira | Low to medium stress |
|  | Paikgacha, Khulna | High stress |
|  | Kalapara, Barguna | Low stress |
|  | Pathorghata, Barguna | Low to medium stress |
| PVT | Tala, Satkhira | Medium stress |
|  | Debhata, Satkhira | Low to high stress |
|  | Kaliganj, Satkhira | High stress |
|  | Dumuria, Khulna | Low stress |
|  | Paikgacha, Khulna | High stress |
|  | Batiaghata, Khulna | Low to medium stress |
|  | Rampal, Bagerhat | Low stress |
|  | Kalapara, Barguna | Low to medium stress |

Q5. Forty to 45 days old seedlings is recommended practice for transplanting in the saline areas? Similarly, Zn dose is also lower than usual recommendation (5kg/ha). Is this Bangladesh specific recommendation?

Response: In Bangladesh, there are three seasons: Aus, Aman and Boro. In Boro seasons, 40 to 45 days-old-seedlings are transplanted in the main field in the coastal saline areas as well. We followed the BRRI recommended doses according to Agro-ecological Zones (AEZ) specificity in Bangladesh. Generally, 10-11 kg ZnSO_4_/ ha was applied during boro season.

Q6. ASV doesn't give exact picture. A good breeding program should look for the YSI (Yield stability index which encompass both stability (ASV) and yield ranking). This can be incorporated quickly as it is a derived parameter but improve and strengthen results for inferences. Please calculate and infer based on YSI. This will improve the confusion on yield data vis-a-vis ASV on page 8.

Response: The lower value of YSI is considered as the most stable with a high grain yield. In this case, G4 and G11 were the most stable genotypes.

Supplementary Table 3: The YSI (Yield stability index), ASV and yield ranking.

| Geno code | Mean Yield | Rank (Yield) | ASV | Rank (ASV) | YSI |
| --- | --- | --- | --- | --- | --- |
| G1 | 5.36 | 9 | 0.50 | 6 | 15 |
| G2 | 4.88 | 14 | 0.57 | 5 | 19 |
| G3 | 4.99 | 13 | 1.13 | 1 | 14 |
| G4 | 6.1 | 3 | 0.87 | 2 | 5 |
| G5 | 5.09 | 10 | 0.34 | 7 | 17 |
| G6 | 5.05 | 12 | 0.15 | 14 | 26 |
| G7 | 5.93 | 5 | 0.14 | 15 | 20 |
| G8 | 5.07 | 11 | 0.16 | 13 | 24 |
| G9 | 5.56 | 8 | 0.30 | 8 | 16 |
| G10 | 5.78 | 7 | 0.26 | 10 | 17 |
| G11 | 6.15 | 2 | 0.71 | 3 | 5 |
| G12 | 6.38 | 1 | 0.26 | 11 | 12 |
| G13 | 4.38 | 15 | 0.65 | 4 | 19 |
| G14 | 5.8 | 6 | 0.27 | 9 | 15 |
| G15 | 6.05 | 4 | 0.24 | 12 | 16 |

Q7. In result table 1, 5 and 6; it would be good if authors could add the salinity stress level against the locations to get the inferences from table immediately. This will remove the confusion to say specific site or trial as stressed, for example selected two genotypes yielded 6.24, 6.34 and 6.36 t/ha comparing to tolerant and sensitive checks (6.41 and 6.01 t/ha respectively). [6 t/ha yield under stress is good yield] - page 12. Two of them are having the potential to produce >7.0 t/ha under favorable - Page 13. How much BR67 and BR28 under favourable envt?

Response: BRRI dhan28 (released in 1994) is a mega variety for Boro season with a limitation of its susceptibility to blast disease and salinity. BRRI dhan67 is another excellent salinity-tolerant variety but its salinity tolerance threshold limit is 8 dS/m in the whole life cycle. Therefore, the development of new varieties (BRRI dhan97 and BRRI dhan99) with more tolerance to salinity like 12 dS/m is a new milestone achievement in salinity breeding.

Q8. Page 13: 2 selected genotypes tolerate 14 dS/m salinity: for how many days comparing to BR67?

Response: The salinity level was observed 14 dS/m consistently at Kaliganj, Satkhira where BRRI dhan97 and BRRI dhan99 were evaluated under PVT. In that situation, both the selected genotypes had survived well and produced considerable yield but BRRI dhan67 (Salt-tolerant check) was completely damaged within 7 days after transplanting as its salinity tolerance level is 8 dS/m (see Figure 5).

Q9. In fig 3, G10 and G4 are on vertices and on positive side of PC1. But you selected G11 and G4 (table 3). G11 is not shown as the winning genotype. Could it be explained, please?

Response: As mentioned earlier, G11 was not the winning genotype in Figure 3, yet it was the top most preferable genotype in the Participatory Varietal Selection (PVS) approach by the farmers of Koyra, Khulna location due to its salinity tolerance, grain quality parameters including long slender grain as well as yield performance.
