# Supplementary material for: Developing climate-resilient rice varieties (BRRI dhan97 and BRRI dhan99) suitable for salt-stress environments in Bangladesh
Source: PLoS One. 2024 Jan 19;19(1):e0294573. doi: 10.1371/journal.pone.0294573 (PMC10810675; doi:10.1371/journal.pone.0294573)
Supplement: Attachment — Submitted filename: Reviewer_2_comments and response.docx [file pone.0294573.s009.docx]

**Reviewer: 2_Comments and Suggestions for Authors**

Reviewer #2: Authors tried to address the issues raised (reviewer 2) but not addressed in the manuscript. I would like to reiterate that we need redressal of points and their inclusion in the main manuscript.

Response: Thank you for the encouraging comments and great suggestions.

Q1. We know the process of varietal release in Bangladesh but it is not known to all the readers. So in the M & M, please include a passing remark in section 2.2 that best performing 12 genotypes are derived from OYT and PYT conducted at XYZ location. If you can, please give the initial numbers of genotypes in OYT and PYT mentioning that: original data on this is not reported, it is acceptable. This way, readers will know that these 12 genotypes are developed through a fixed process (not arbitrary).

Response: In the manuscript, we included the varietal release system in Bangladesh as Table 1 and the background history of the studied genotypes in the M & M (section 2.2) as well as OYT and PYT conducted at the different locations.

Q2. When i mentioned that please avoid giving grey literature or not use it unless very authentic/related reference, authors responded with citations from authorities like Epstein but not included into the manuscript. Why? Just to convince me? We need to convince readers. Can a paper on "Plant Growth Promoting" bacteria substantiate it, that too a very low-ranking journal? Probably authors could not understand what i wanted to convey. Please do the needful.

Response: We addressed your suggestions and cited relevant references in the manuscript.

Q3. About my query number 4 on stress status of trail locations. Again Authors tried to respond me through a very vague table. It should be very precise or with a range of stress. What is low, medium, high, low to high? Can authors mention some range at least and give as supplementary table to strengthen 2.1 section? It should be for all readers (not only for me).

Response: We made a table containing the trials, locations, salinity status, and ranges (minimum to maximum). We attached this table in the main manuscript as a Table 1. Besides, we also attached soli salinity classes and the ranges of Electrical Conductivity (EC) as a Supplementary Table 3.

Table 1: The characterization of the studied areas including trials, locations, salinity status, and salinity ranges (minimum to maximum)

| Trials | Location name | Status of salinity | Ranges (dS/m) |
| --- | --- | --- | --- |
| RYT | Assasuni, Sathkira | Low stress (Non saline to very slight saline) | 3.73-5.25 |
|  | Kaliganj, Satkhira | High stress (very slight saline to moderate saline) | 3.96-15.01 |
|  | Debhata, Satkhira | Low to high stress (very slight saline to slight saline) | 2.47-6.99 |
|  | Koyra, Khulna | Medium stress (slight saline to moderate saline) | 7.85-13.20 |
| ALART | BRRI Gazipur | No stress (Favorable) |  |
|  | Debhata, Satkhira | Low to high stress (very slight saline to slight saline) | 3.25-7.09 |
|  | Assasuni, Satkhira | Low stress (Non saline to very slight saline) | 3.90-6.05 |
|  | Batiaghata, Satkhira | Low to medium stress (very slight saline to slight saline) | 2.70-7.35 |
|  | Paikgacha, Khulna | High stress (very slight saline to moderate saline) | 4.13-11.58 |
|  | Kalapara, Barguna | Low stress (Very slight saline to slight saline) | 3.53-7.01 |
|  | Pathorghata, Barguna | Low to medium stress (very slight saline to slight saline) | 3.21-7.83 |
| PVT | Tala, Satkhira | Medium stress (Very slight saline to slight saline) | 4.07-8.17 |
|  | Debhata, Satkhira | Low to high stress (very slight saline to moderate saline) | 3.10-12.29 |
|  | Kaliganj, Satkhira | High stress (very slight saline to strong saline) | 3.55-16.17 |
|  | Dumuria, Khulna | Low stress (Very slight saline to slight saline) | 3.61-6.79 |
|  | Paikgacha, Khulna | High stress (Very slight saline to moderate saline) | 4.23-11.79 |
|  | Batiaghata, Khulna | Low to medium stress (very slight saline to slight saline) | 3.40-8.50 |
|  | Rampal, Bagerhat | Low stress (Very slight saline to slight saline) | 4.08-7.53 |
|  | Kalapara, Barguna | Low to medium stress (very slight saline to slight saline) | 3.93-6.99 |

Supplementary Table 3: Soli salinity classes and the range of Electrical Conductivity (EC) (**Source: Soil Resource Development Institute, 2010)**

| Salinity class | EC (dSm^-1^) |
| --- | --- |
| Non-saline to very slight saline | 2-4 |
| Very slight saline to slight saline | 4-8 |
| slight saline to moderate saline | 8-12 |
| Moderate saline to strong saline | 12-16 |
| strong saline to very strong saline | >16 |

Q4. About my query 5: there is something wrong with Zn calculation. You mentioned 10-11 kg ZnSO4/ha. But the sentence in section 2.3 says: "Fertilizers were supplied at a rate of 120:19:60: 20:3.6 kg NPKSZn/ha (260-97-120-110-11kg/ha, respectively in the form of urea, TSP, MoP, gypsum, and zinc sulphate)". The minimum Zn is 20% in ZnSO4 and once Zn is heptahydrate or so, the concentration varies. So 3.6 Zn cannot be 11 kg ZnSO4, by any means. So please confirm if 3.6 is correct or 11 kg is correct.

Response: 11 kg is correct and Zn content is also corrected in the section 2.3 of the Manuscript "Fertilizers were supplied at a rate of 120:19:60: 20:4.01 kg NPKSZn/ha (260-97-120-110-11kg/ha, respectively in the form of urea, TSP, MoP, gypsum, and zinc sulphate)".

Q5. About query 6 on YSI, i did not find any mention of YSI calculation in M & M. Please include its derivation. When Fig 3 is not able to conclusively select G11; and your selection is based on YSI for G4 and G11; why you donot include the YSI supplementary table 3 as main table in the text. Please do so as this table is the concrete basis of your genotype selection.

Response: Yield stability index (YSI) derivation is attached in the M & M section (2.5) and Yield Rank (RYi), ASV Rank (RASVi), and YSI value are included in the manuscript as a table (Table 4).

Yield stability index (YSI) for individual genotypes which combines both mean grains yield and ASV index was estimated as YSI = RASVi + RYi. Here, the rank of the AMMI stability value for the ith genotype is RASVi, while the rank of the mean grain yield for the ith genotype across environments is RYi.

Included in Table 4: The YSI (Yield stability index), ASV and yield ranking.

| Geno code | Mean Yield | ASV | Yield Rank (RYi) | ASV Rank (RASVi) | YSI |
| --- | --- | --- | --- | --- | --- |
| G1 | 5.36 | 0.50 | 9 | 6 | 15 |
| G2 | 4.88 | 0.57 | 14 | 5 | 19 |
| G3 | 4.99 | 1.13 | 13 | 1 | 14 |
| G4 | 6.1 | 0.87 | 3 | 2 | 5 |
| G5 | 5.09 | 0.34 | 10 | 7 | 17 |
| G6 | 5.05 | 0.15 | 12 | 14 | 26 |
| G7 | 5.93 | 0.14 | 5 | 15 | 20 |
| G8 | 5.07 | 0.16 | 11 | 13 | 24 |
| G9 | 5.56 | 0.30 | 8 | 8 | 16 |
| G10 | 5.78 | 0.26 | 7 | 10 | 17 |
| G11 | 6.15 | 0.71 | 2 | 3 | 5 |
| G12 | 6.38 | 0.26 | 1 | 11 | 12 |
| G13 | 4.38 | 0.65 | 15 | 4 | 19 |
| G14 | 5.8 | 0.27 | 6 | 9 | 15 |
| G15 | 6.05 | 0.24 | 4 | 12 | 16 |

Here, RASVi = Rank of the AMMI stability value for the ith genotype, RYi = Rank of the mean grain yield for the ith genotype across environments

Q6. With reference to my query 7, you did not address what i wanted to do. But if you don't want include the stress level in table 1, 5 and 6 (but it would be better if you do); please respond to my above-mentioned query no.3 with definite range.

Response: We have already mentioned (query no.3) the salinity ranges and status of the different trials and studied locations are given as a manuscript and supplementary (Table1 & Supplementary Table 3).

Q7. Few small corrections: under section 2.7 (Chemical property), another abbreviation of ASV used. Please highlight this in the section that it is an alkali spreading value for grain quality trait. Otherwise, the reader may be confused. Another correction in table 3, column 6, 7 and 8 are for grain yield at XYZ location. Please correct it.

Response: We corrected the alkali spreading value (ASV) in section 2.7 for chemical properties. Another in Table 4 is corrected by Assasuni (E1) yield (tha^-1^), Debhata (E2) yield (tha^-1^) and Koyra (E3) yield (tha^-1^).
